# Development of an interpretable model for foot soft tissue stiffness based on gait plantar pressure analysis

**DOI:** 10.3389/fbioe.2024.1482382

**Published:** 2025-01-06

**Authors:** Xiaotian Bai, Xiao Hou, Dazhi Lv, Jialin Wei, Yiling Song, Zhengyan Tang, Hongfeng Huo, Jingmin Liu

**Affiliations:** ^1^ Department of Physical Education, Tsinghua University, Beijing, China; ^2^ School of Sport Science, Beijing Sport University, Beijing, China; ^3^ College of Physical Education, Hebei Normal University, Shijiazhuang, China; ^4^ Key Laboratory of Bioinformatics Evaluation of Human Movement, Hebei Normal University, Shijiazhuang, China

**Keywords:** neural network, plantar soft tissue, gait, plantar pressure, biomechanics

## Abstract

**Purpose:**

Plantar soft tissue properties affect foot biomechanics during movement. This study aims to explore the relationship between plantar pressure features and soft tissue stiffness through interpretable neural network model. The findings could inform orthotic insole design.

**Methods:**

A sample of 30 healthy young male subjects with normal feet were recruited (age 23.56 ± 3.28 years, height 1.76 ± 0.04 m, weight 72.21 ± 5.69 kg). Plantar pressure data were collected during 5 trials at the subjects’ preferred walking speed (1.15 ± 0.04 m/s). Foot soft tissue stiffness was recorded using a MyotonPRO biological soft tissue stiffness meter before each walking trial. A backpropagation neural network, optimized by integrating particle swarm optimization and genetic algorithm, was constructed to predict foot soft tissue stiffness using plantar pressure data collected during walking. Mean impact value analysis was conducted in parallel to investigate the relative importance of different plantar pressure features.

**Results:**

The predicted values for the training set are slightly higher than the actual values (MBE = 0.77N/m, RMSE = 11.89 N/m), with a maximum relative error of 7.82% and an average relative error of 1.98%, and the predicted values for the test set are slightly lower than the actual values (MBE = −4.43N/m, RMSE = 14.73 N/m), with a maximum relative error of 7.35% and an average relative error of 2.55%. Regions with highest contribution rates to foot soft tissue stiffness prediction were the third metatarsal (13.58%), fourth metatarsal (14.71%), midfoot (12.43%) and medial heel (12.58%) regions, which accounted for 53.3% of total contribution.

**Conclusion:**

The pressure features in the medial heel, midfoot area, and lateral mid-metatarsal regions during walking can better reflect plantar soft tissue stiffness. Future studies should ensure measurement stability of this region and refine insole designs to mitigate plantar soft tissue fatigue in the specified areas.

## 1 Introduction

Plantar soft tissue is an important structure for maintaining foot mobility function, and its mechanical properties affect foot performance during motion (Mckeon et al., 2015; Lynn et al., 2012; Natali et al., 2010; Ker et al., 1987). The plantar soft tissue consists of fat pads, fascia, muscles and tendons in the sole of the foot. Due to its viscoelasticity, the plantar soft tissue can coordinate the rigid structures of the foot to adapt to different modes of movement. In the plantar soft tissue, the fat pad in the heel can provide cushioning and protect the bones and joints during movement (Mckeon et al., 2015; Teng et al., 2022); the muscles in the sole can increase the rigidity of the foot to provide power for improved locomotion efficiency (Ridola and Palma, 2001); the plantar fascia can store energy during weight bearing, improving locomotion economy through its viscoelasticity (Natali et al., 2010; Ker et al., 1987). Studies show that the stiffness of the proximal plantar fascia increases with greater dorsiflexion angles and tension in the Achilles tendon (Liu et al., 2020; Shiotani et al., 2023), and the stiffness of the Achilles tendon and plantar fascia are important indicators for assessing plantar fasciitis (Bolivar et al., 2013; Baur et al., 2021). Investigations of the plantar fascia in overweight and obese groups showed reduced stiffness of the muscular fascia in the sole of the foot relative to normal weight populations (Tas et al., 2017), and decreased rigidity of the plantar fascia may affect midfoot stability, leading to excessive pronation (Cifuentes-De et al., 2021; Huang et al., 1993). Moreover, diabetic foot patients have higher stiffness than healthy people in the plantar soft tissue (Chatzistergos et al., 2014). Behforootan et al. believe that understanding the stress-strain capabilities of plantar soft tissue under daily weight-bearing conditions is important for understanding the etiology of foot ulcers (Behforootan et al., 2017). It can be seen that the soft tissues of the sole play an important role in improving the adaptability and economy of movement of the foot during exercise. Exploring the biomechanical performance of the foot during exercise is an important reference for reflecting the state of the soft tissues of the sole.

Current methods for measuring plantar soft tissue stiffness can be divided into two categories: ultrasound technology and non-invasive physical detection techniques. Ultrasound-based devices include ultrasound diagnostic instruments and ultrasound elastography equipment. The principle is to evaluate plantar soft tissue stiffness by calculating shear modulus based on the velocity attenuation of ultrasound waves in plantar soft tissues (Baur et al., 2021; Gatz et al., 2020). Non-invasive physical detection techniques mainly apply mechanical pressure on plantar soft tissues and measure tissue rebound or feedback to quantify plantar soft tissue stiffness. Devices using this technique include MyotonPRO biological soft tissue stiffness meter and some custom-designed plantar pressure devices by researchers (Chatzistergos et al., 2014; Sakalauskaitė and Satkunskienė, 2012; Teoh and Lee, 2016). However, the aforementioned test methods can only measure plantar soft tissue stiffness under non-weight-bearing and static conditions, lacking integration with actual physical activity, and many foot soft tissue diseases often arise from the foot repeatedly buffering, propelling, walking and other functions while bearing body weight. Pathological changes in the force characteristics of different plantar regions during walking often lead to changes in plantar soft tissue stiffness, thus resulting in various plantar soft-tissue related diseases (such as plantar fasciitis, heel pain, diabetic foot, etc.) (Bolivar et al., 2013; Chatzistergos et al., 2014; Cheung et al., 2006). Finite element analysis indicates that softening plantar tissues in pes cavus can reduce stress on metatarsals, thereby mitigating metatarsalgia (Cen et al., 2023). The aforementioned content underscores the necessity of identifying key plantar regions that exhibit the functional characteristics of soft tissues during physical activity. This is instrumental in enhancing the design of foot orthoses and in formulating appropriate intervention strategies for the plantar soft tissues.

Although traditional analytical methods (such as correlation analysis) can explore associations between variables, they are typically limited to linear relationship analysis between single variables. In contrast, the neural network is more suitable for the regression problem, which involves a relatively small number of input parameters, and can demonstrate rapid training speed and good convergence performance. This study selected the average pressure of different plantar regions during the walking stance phase as the input layer, and tested the *in vivo* plantar soft tissue stiffness of the subjects using a MyotonPRO biological soft tissue testing device as the output layer. Optimizing a Backpropagation (BP) Neural Network through Particle Swarm Optimization (PSO) and Genetic Algorithms (GA), in conjunction with the Mean Impact Value (MIV) method, to explore the relationship between the stiffness of the plantar soft tissues and the mechanical characteristics of the plantar region during walking.

## 2 Methods

### 2.1 Subjects

Referring to previous studies on plantar fascia (Sakalauskaitė and Satkunskienė, 2012; Huang et al., 2018), 30 young male participants were recruited for this study, with the following inclusion criteria: (1) participants exhibited normal foot arch types (with the arch index between 0.21 and 0.26 (Cavanagh and Rodgers, 1987)); (2) no history of lower limb or plantar fascia injuries in the past 6 months; (3) no engagement in intense physical activity within 48 h prior to the study. The basic information of the subjects is presented in Table 1. The testing procedures were explained to all subjects and informed consent was obtained prior to testing. This study was approved by the Ethics Committee of Hebei Normal University (No. 2022LLSC026), and the elements of the study involving human research were conducted in accordance with the Declaration of Helsinki.

**TABLE 1:** Basic information of the subjects.

	Age (years)	Height(m)	Weight (kg)	Arch index
Value	23.56 ± 3.28	1.76 ± 0.04	72.21 ± 5.69	0.23 ± 0.02

### 2.2 Data collection

#### 2.2.1 Plantar pressure data acquisition

Plantar pressure data during walking was collected using a high frequency plantar pressure plate (RSscan International, Belgium, sampling frequency 120Hz, minimum resolution 0.25N, measurement range 1–60N/cm^2^, plate length 2 m). Extensions of 1.5 m were added at the beginning and end of the pressure plate to record changes in plantar loading during progression. Under the guidance of the experimenters, all participants first completed the same warm-up routine, which involved mobilizing the knee and ankle joints and stretching the thigh and calf muscles, then walked 2-3 times over the pressure plate to get familiar with the testing protocol. Plantar pressure data of 5 trials under each subject’s preferred walking speed (1.15 ± 0.04 m/s) were recorded, with at least one left and one right step in each trial.

#### 2.2.2 Plantar soft tissue stiffness data acquisition

**FIGURE 1:**
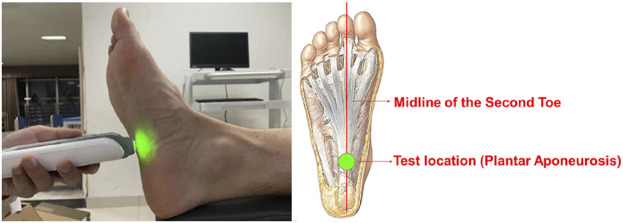
Plantar soft tissue stiffness testing.

After subject warm-up, a MyotonPRO biological soft tissue stiffness meter (Myoton AS, Estonia, measurement depth 20–30mm, stiffness range 70–1900N/m, coefficient of variation 1.7%, acceleration resolution ±8 g) was used to record plantar soft tissue stiffness. Subjects lied in a supine relaxed position with the ankle and metatarsophalangeal joints aligned in a neutral position (Chatzistergos et al., 2014; Behforootan et al., 2017). The probe was placed perpendicular to the intersection of the anterior calcaneus and second toe midline (plantar aponeurosis location (Sakalauskaitė and Satkunskienė, 2012)) until the green light turned on (Figure 1). The tester held the device steadily at the measuring position to collect data. Both feet were measured 5 times for each subject, each test was conducted prior to the walking test to ensure that the plantar soft tissue stiffness data corresponded with the walking data.

### 2.3 Data processing

#### 2.3.1 Plantar pressure data processing

The average pressure of ten plantar regions was calculated using Equation 1:

$$F_{mean}=\frac{\sum F_{i}}{n}$$ (1)

(where $F_{mean}$ is the average plantar pressure, $F_{i}$ is the pressure of that region at the *ith* sampling, and n is the total number of samples).

**FIGURE 2:**
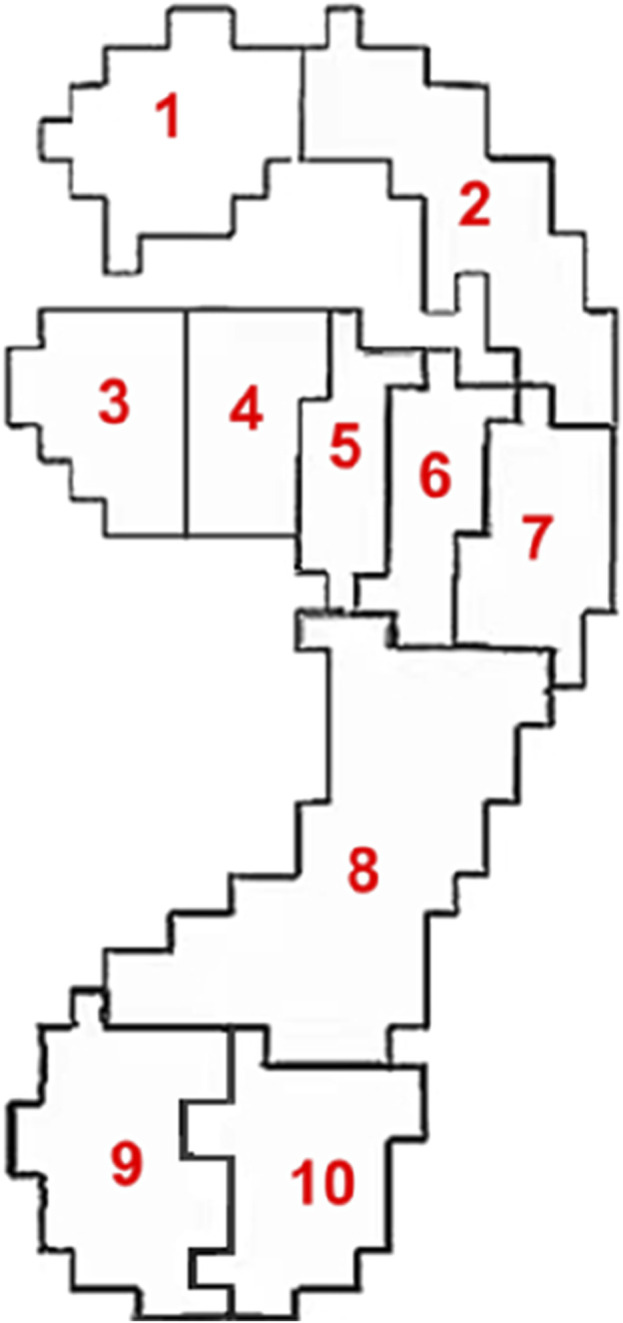
Schematic diagram of plantar regions.

The ten plantar regions were divided according to the Footscan software included with the high frequency plantar pressure plate. The division of plantar regions is shown in Figure 2, where region one is the hallux, region two is the second to fifth toes, regions three to seven are the first to fifth metatarsals respectively, region eight is the midfoot, region nine is the medial heel and region 10 is the lateral heel.

#### 2.3.2 Plantar soft tissue stiffness data processing

**FIGURE 3:**
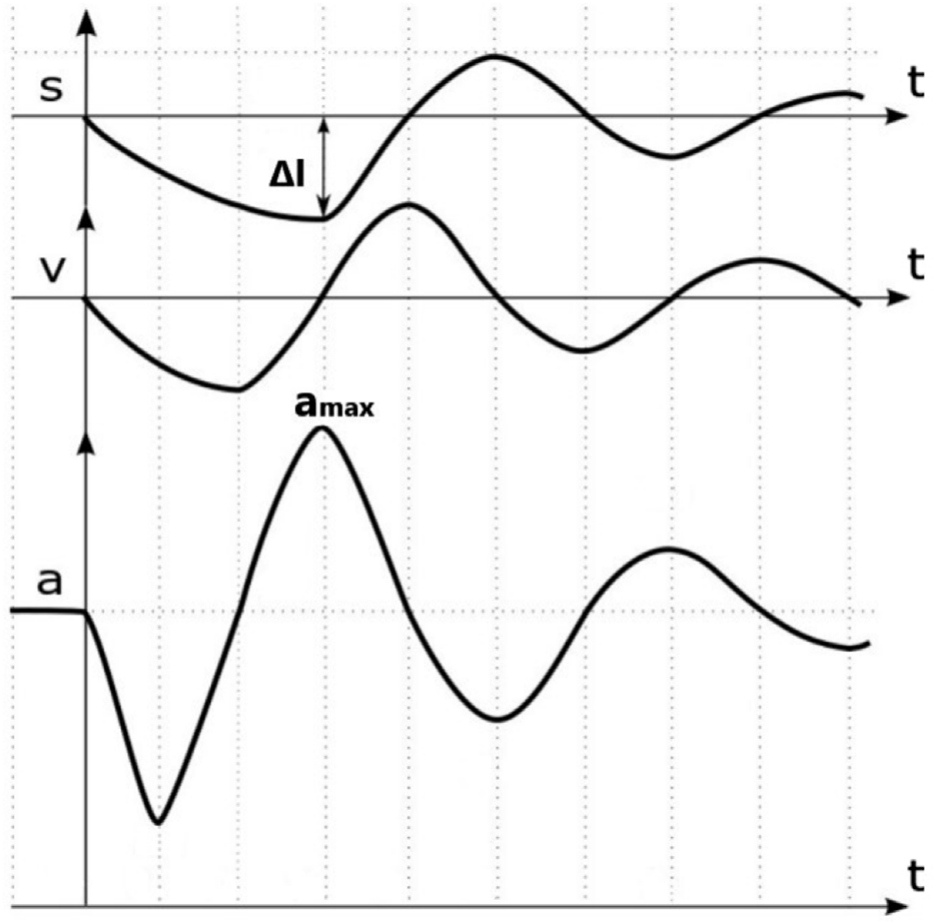
Example of test data. (Note: s is the deformation variable, v is velocity, and a is acceleration).

The MyotonPRO biological soft tissue stiffness meter mainly records the damped natural oscillation of soft tissues in the form of acceleration signals. It calculates soft tissue stiffness from the force of the probe and deformation of the soft tissue. An example of the test data is shown in Figure 3. Soft tissue stiffness was calculated using Equation 2:

$$S=\frac{a_{\max}\cdot m_{probe}}{∆l}$$ (2)

(where S is the soft tissue stiffness, $a_{\max}$ is the maximum acceleration value, $m_{probe}$ is the probe mass, and ∆l is the soft tissue deformation variable).

#### 2.3.3 Neural network construction

**FIGURE 4:**
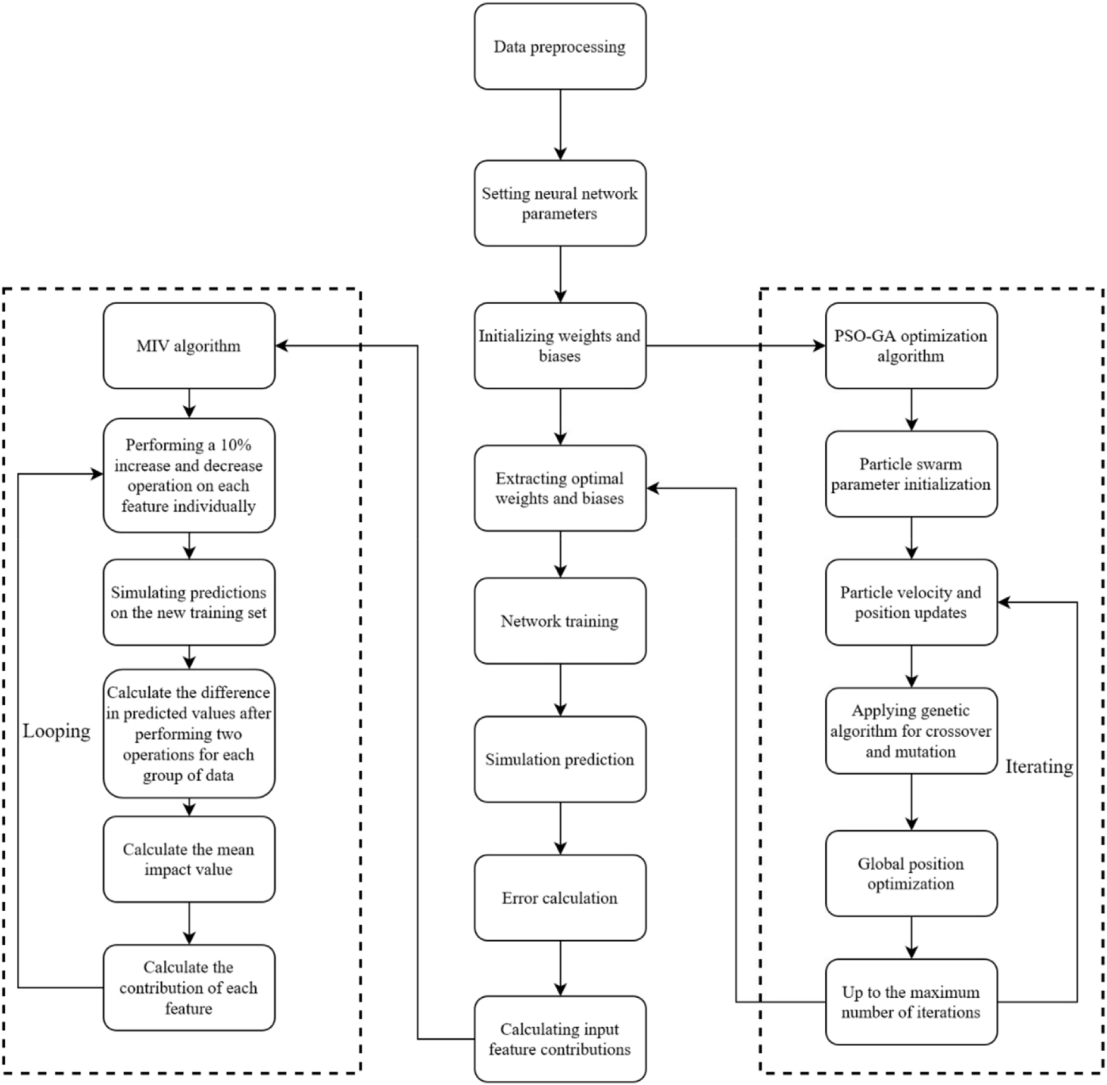
Neural network construction.

The neural network was constructed using the Neural Network Toolbox in MATLAB 2022b, combined with open-source PSO and GA code from Github. The workflow is shown in Figure 4. The specific steps are as follows.

##### 2.3.3.1 Data preprocessing

The plantar pressure data from five successful walking trials for both feet of the 30 subjects was constructed into a 10 × 300 input layer. The corresponding plantar soft tissue stiffness from each walking trial was constructed into a 1 × 300 output layer. To improve the stability and accuracy of the neural network, the input and output layer data were normalized to the range (0, 1) respectively to accelerate convergence (Xu et al., 2021), as shown in Equation 3.

$$x_{standard}=\frac{x_{i}-x_{\min}}{x_{\max}-x_{\min}}$$ (3)

(where $x_{standard}$ is the normalized data, $x_{\min}$ is the minimum value in the target dataset, and $x_{\max}$ is the maximum value in the target dataset).

##### 2.3.3.2 Setting neural network parameters

A three-layer multi-input single-output BP regression neural network was constructed with 10 nodes in the input layer, 13 nodes in the hidden layer (determined by Equation 4, (Gu et al., 2020; Xia et al., 2019)), and one node in the output layer. The network was set to train for 8,000 iterations, with a target error of 1 × 10^−6^ and a learning rate of 0.01.

$$Hidden=\sqrt{Input+Output}+a$$ (4)

(where Hidden is the number of hidden layer nodes, Input is the number of input layer nodes, Output is the number of output layer nodes, and a is a constant between Equations 1, 9).

##### 2.3.3.3 Initializing weights and biases

The PSO-GA optimization algorithm was used to initialize the neural network weights and biases. During each iteration of the PSO, the best individual identified by the genetic algorithm is used to replace the worst individual in the PSO population. This strategy harnesses the complementary strengths of both PSO and GA, enhancing overall optimization performance. The main parameters of the PSO-GA optimization algorithm are shown in Table 2. After each update, the fitness value of each particle was calculated (Gu et al., 2020; Xia et al., 2019) using Equation 5.

$$Fitness=\sum_{m=1}^{n_{t}}\sqrt{{\frac{1}{n_{t}}\left(t_{m}-\hat{t_{m}}\right)}^{2}}$$ (5)

(where a Fitness is the fitness value, $n_{t}$ is the training dataset size, $t_{m}$ is the true value for the *m*th group of training data, and $\hat{t_{m}}$ is the predicted value for the *m*th group of training data).

**TABLE 2:** Main parameters of the PSO-GA optimization algorithm.

PSO				GA			
Iteration	Individual learning factor	Population learning factor	Swarm size	Generation	Swarm size	Accuracy	Crossover rate
30	4.5	4.5	5	50	5	1 × 10^−6^	0.4

##### 2.3.3.4 Extracting optimal weights and biases

The convergence of the algorithm was evaluated by plotting the change in fitness values over each PSO-GA iteration. After all PSO-GA iterations were complete, the particle position with the minimum fitness value was selected, containing the optimal weights and biases.

##### 2.3.3.5 Network training

The optimal weights and biases were assigned to the connection weights and biases of the neural network. The training and test sets were divided in a 8:2 ratio for network training.

##### 2.3.3.6 Simulation prediction

The optimized neural network was used to perform simulation predictions on the test and training sets, followed by inverse normalization of the data.

##### 2.3.3.7 Error calculation

Both absolute and relative error metrics were employed to evaluate the model performance on the training and test sets. For absolute errors, the mean bias error (MBE) and root mean square error (RMSE) were calculated as presented in Equations 6, 7, (Xu et al., 2021; Li et al., 2022). For relative errors, the relative error percentage (REP) was computed as presented in Equation 8, (Xu et al., 2021; Li et al., 2022).

$$MBE=\frac{\sum_{m=1}^{n}\left(y_{m}-{\hat{y}}_{m}\right)}{n}$$ (6)

$$RMSE=\sqrt{\frac{1}{n}\sum_{m=1}^{n}{\left(y_{m}-{\hat{y}}_{m}\right)}^{2}}$$ (7)

$$REP=\frac{\left|y_{m}-{\hat{y}}_{m}\right|}{y_{m}}\times 100\%$$ (8)

(Where n is the dataset size, $y_{m}$ is the true value for the *m*th data, and ${\hat{y}}_{m}$ is the predicted value for the *m*th data).

##### 2.3.3.8 Calculating input feature contributions

The MIV algorithm was used to calculate the contribution of each input feature to the output. The method begins by perturbing each input variable by ±10%, after which the trained neural network is used to predict the outputs for the perturbed data, generating new results. By comparing the output changes before and after the perturbation, the MIV is calculated. A positive MIV indicates the input feature has a positive influence on the output, while a negative MIV means the input has a negative influence on the output (Gu et al., 2020; Xia et al., 2019; Li et al., 2022).

$$MIV=\frac{P_{i1}-P_{i2}}{n}$$ (9)

(Where $P_{i1}$ is the predicted data after increasing the *i*th feature by 10%, $P_{i2}$ is the predicted data after decreasing the *i*th feature by 10%, and n is the data size).

The MIV value for each feature was calculated according to the above steps. The absolute MIV value for each feature was divided by the sum of absolute MIV values for all features to obtain the contribution rate of each feature (Gu et al., 2020; Xia et al., 2019; Li et al., 2022), as presented in Equation 10:

$${Con}_{i}=\frac{\left|{MIV}_{i}\right|}{\sum_{i=1}^{10}{MIV}_{i}}\times 100\%$$ (10)

(Where ${Con}_{i}$ is the contribution rate of the *i*th feature, and ${MIV}_{i}$ is the mean impact value of the *i*th feature).

### 2.4 Statistical analysis

The data collected in this study were processed and statistically analyzed by Excel and SPSS25.0. The neural network construction, training, and error calculation were performed in Matlab 2022b.

## 3 Results

### 3.1 Evaluation of neural network performance

#### 3.1.1 Results of optimization algorithm

**FIGURE 5:**
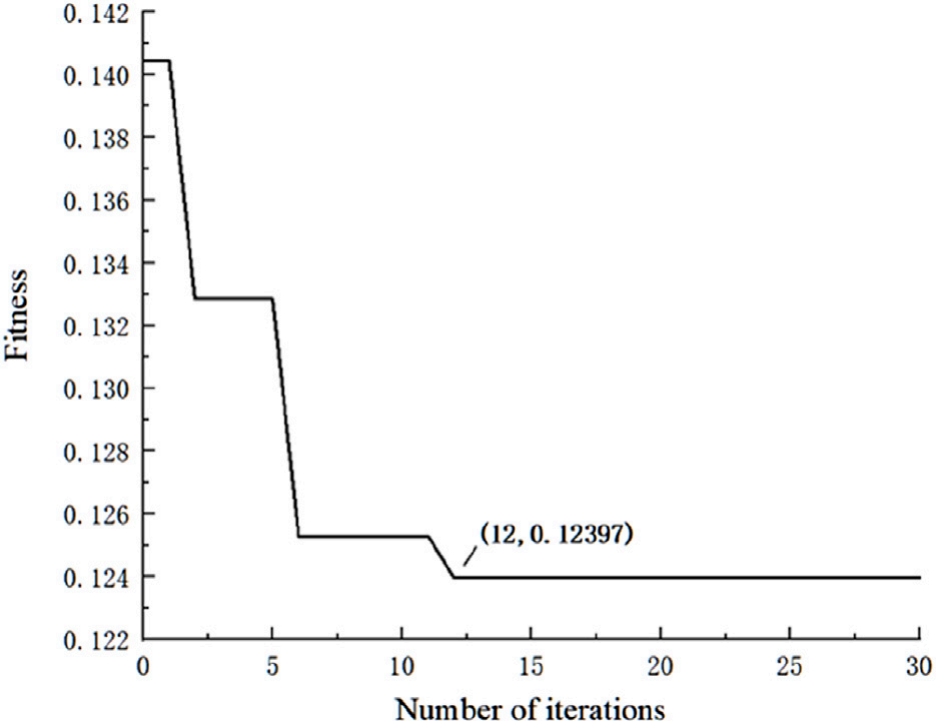
Changes in fitness during training.

The PSO-GA optimization convergence curve is shown in Figure 5. The fitness value stabilized around the 12th iteration, indicating that the network reached convergence with an optimal fitness value of 0.12397.

#### 3.1.2 Results of Model Prediction Performance

**FIGURE 6:**
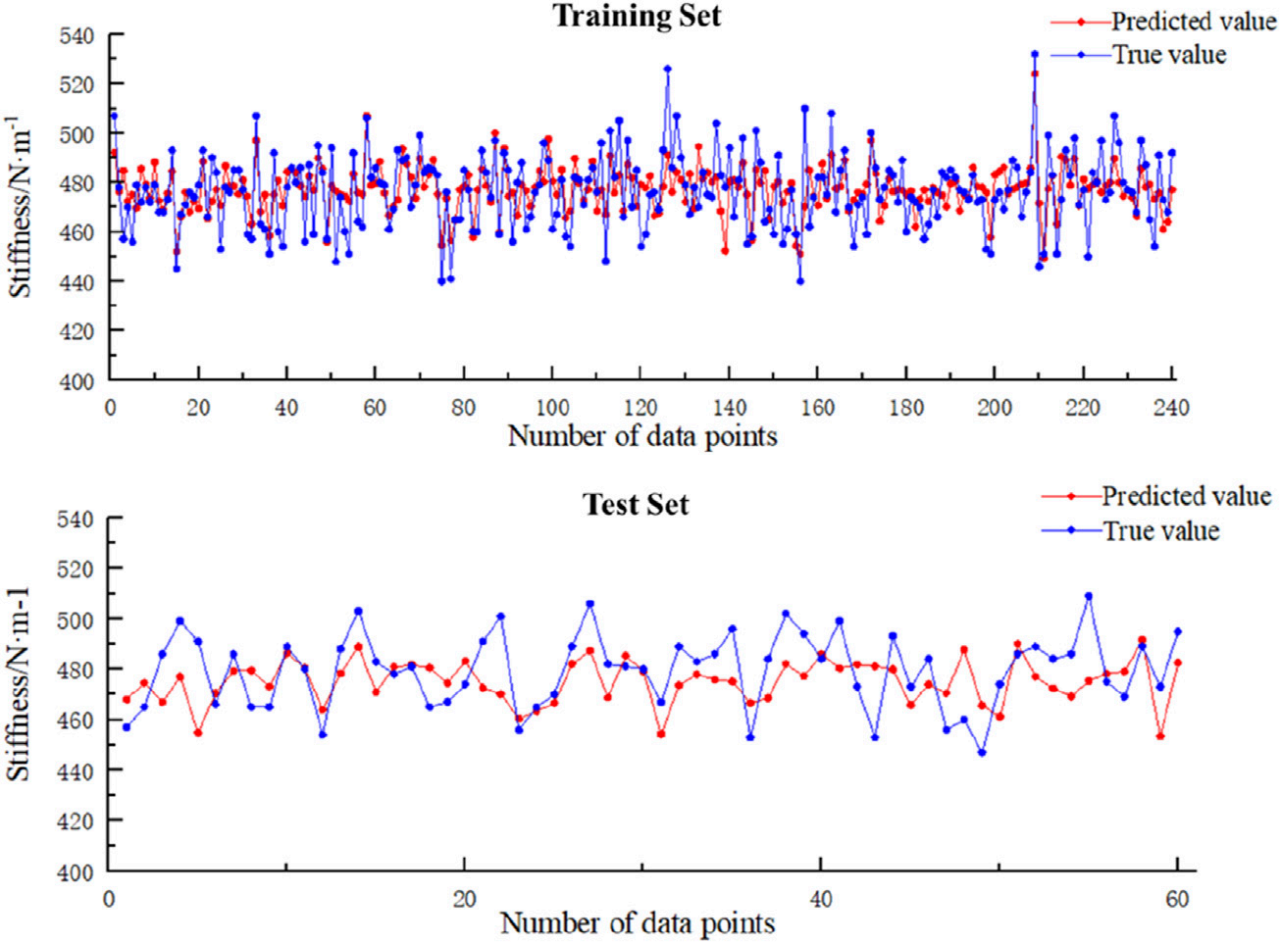
Model prediction performance.

As shown in Table 3 and Figure 6, the predicted values for the training set are slightly higher than the actual values (MBE = 0.77N/m, RMSE = 11.89 N/m), with a maximum relative error of 7.82% and an average relative error of 1.98%, and the predicted values for the test set are slightly lower than the actual values (MBE = −4.43N/m, RMSE = 14.73 N/m), with a maximum relative error of 7.35% and an average relative error of 2.55%.

**TABLE 3:** Error calculation results of the training and test sets.

	Absolute error		Relative error	
	MBE (N/m)	RMSE (N/m)	Maximum REP (%)	Average REP (%)
Training set	0.77	11.89	7.82	1.98
Test set	−4.43	14.73	7.35	2.55

### 3.2 Contribution rates of input features

**FIGURE 7:**
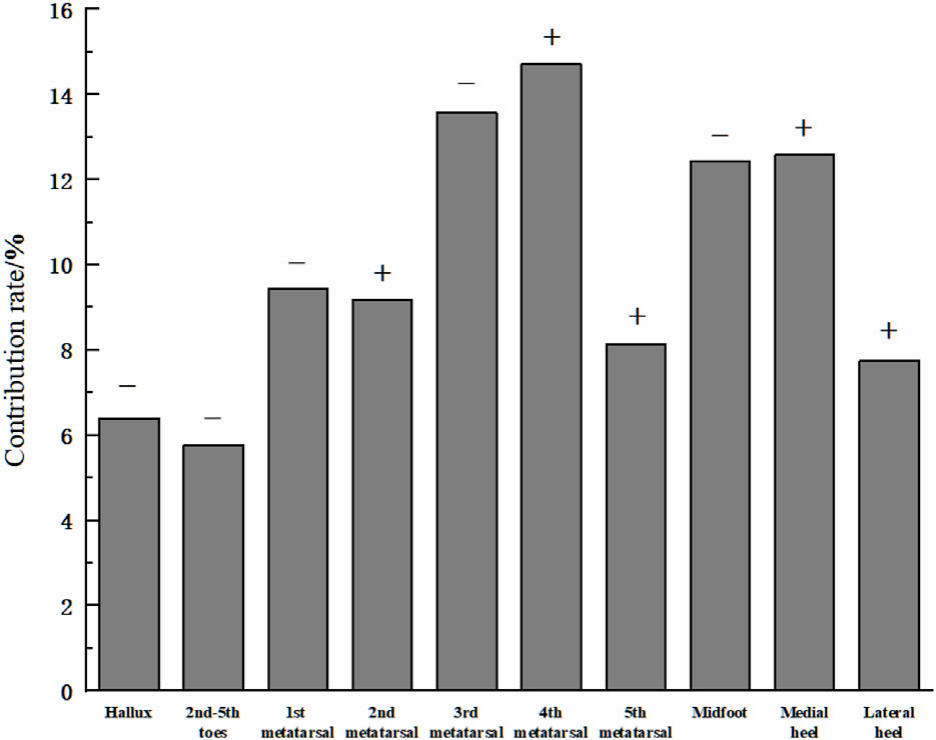
Contribution rates of input features. (Note: “+” indicates a positive influence of the input feature on the output. “-” indicates a negative influence).

The contribution rates of each input feature to the output are shown in Figure 7. As illustrated, the average pressure of the third metatarsal (13.58%), fourth metatarsal (14.71%), midfoot (12.43%), and medial heel (12.58%) regions accounted for over 10% each, comprising 53.3% of the total contribution. MIV results indicate that the average pressure in the hallux (6.41%), second-fifth toes (5.78%), first metatarsal (9.44%), and midfoot (12.43%) regions had a negative influence on plantar soft tissue stiffness. In contrast, the average pressure of the second metatarsal (9.17%), fourth metatarsal (14.71%), fifth metatarsal (8.15%), medial heel (12.58%), and lateral heel (7.75%) regions positively influenced plantar soft tissue stiffness.

## 4 Discussion

This study used the average pressure of each plantar region during the stance phase of walking as the input layer and the plantar soft tissue stiffness under the plantar fascia in a non-weight-bearing state as the output layer. By combining actual motion in daily life (i.e., walking) to optimize the neural network algorithm, the relationship between static stiffness and dynamic plantar mechanics during foot motion was explored. The results showed that the average pressure during walking in the third metatarsal region (13.58%), fourth metatarsal region (14.71%), midfoot region (12.43%) and medial heel region (12.58%) made relatively vital contributions to plantar soft tissue. Among them, the third metatarsal and midfoot regions had positive effects on plantar soft tissue stiffness, while the fourth metatarsal region and medial heel region had negative effects.

### 4.1 Discussion of neural network performance

This study develops an interpretable neural network to extract plantar pressure indicators that reflect the condition of soft tissues. To improve the accuracy of the model, a larger dataset was used, with an 8:2 split between the training and test sets. Consequently, enabled the application of machine learning techniques to more effectively capture the key indicators in the input layer. For the output layer, soft tissue stiffness of the rearfoot region was measured by MyotonPRO, which obtained the result of 476.99 ± 15.26 N/m in this study, similar to the findings by Huang et al. and Sakalauskaite et al. using the same device (Sakalauskaitė and Satkunskienė, 2012; Huang et al., 2018), indicating favorable stability of the measurements in this study. During network training, fitness was calculated in each iteration using the root mean square error between predicted and actual values of the training set. As shown in Figure 5, at the 12th iteration the fitness value of optimization algorithm converged and remained below 0.124. Comparing the errors of the training and testing sets showed the overall maximum REP did not exceed 8% with an average REP below 3%. Concurrently, the RMSEs for the training and testing sets were less than 15% with average absolute errors below 13 N/m. It demonstrates that the PSO-GA-BP regression network can effectively predict plantar soft tissue physical properties, which achieving prediction of plantar soft tissue physical properties through biomechanical characteristics of the foot during daily exercise. In addition, the key plantar areas reflecting soft tissue stiffness during walking can be analyzed based on the constructed neural network.

### 4.2 Discussion of plantar mechanics and soft tissue stiffness

The plantar soft tissue stiffness is an important parameter for evaluating foot function and diagnosing foot disorders (Cifuentes-De et al., 2021; Chatzistergos et al., 2014; Teoh and Lee, 2016). This study utilized MIV to quantify feature importance in neural networks, identifying the third and fourth metatarsals, midfoot, and lateral heel as critical indicators of plantar soft tissue biomechanics. During walking, the heel cushions impact via the plantar fat pad. Higher impact frequency and force indicate more rigid buffering during the heel strike phase (Bai et al., 2022). Research has shown that narrower and higher heels increase plantar fascia stress, while lower heel heights contribute to alleviating tension in plantar soft tissues (Wang et al., 2021). Our study found the average pressure in both medial and lateral heel correlated positively with plantar soft tissue stiffness. This suggests that incorporating enhanced heel cushioning in footwear design may help alleviate stiffness in the plantar fascia. Matthew R et al. also found that using calcaneal taping to prevent excessive eversion of the calcaneus can help support the function of the medial longitudinal arch, thereby reducing tension on the plantar fascia and alleviating heel pain (Hyland et al., 2006). These outcomes indicate individuals with poorer heel cushioning or abnormal calcaneus position may have stiffer plantar soft tissues. It is recommended to incorporate both enhanced calcaneal cushioning and stabilization features in footwear design to help relieve pressure on the plantar fascia.

For the midfoot region, Peng et al. used a musculoskeletal flatfoot model and found arch-supporting insoles can effectively reduce peak plantar pressure and strain in the plantar fascia (Peng et al., 2022). Our results also showed the average midfoot pressure negatively influenced plantar soft tissue stiffness, indicating that appropriately raising the arch height to increase midfoot pressure may relieve stiffness of the plantar fascia, consistent with Peng’s findings. Although existing research on flatfoot has only found thicker plantar fascia without differences in stiffness compared to normal feet (Tas et al., 2018), some studies show higher incidence of plantar fasciitis in flatfoot patients (Huang et al., 2004; Lee et al., 2023). Researches have also shown that elevating the arch in flat-footed patients not only helps distribute plantar pressure more evenly, but also improves foot comfort (Xu et al., 2019; Kasai et al., 2024). For the general population, a moderate elevation of the arch can enhance comfort as well (Lewin and Price, 2024). These findings suggest that adjusting the load-bearing pattern of the arch may help alleviate tension in the plantar fascia.

In the forefoot, the windlass mechanism is used during walking to increase arch stiffness and enable the rigid lever function of the foot for push-off (Kelly et al., 2014). In this study, the third and fourth metatarsals showed high contribution to plantar soft tissue stiffness, reflecting their important role in push-off. However, their influence on stiffness was opposite during walking, with lateral metatarsals (fourth and fifth) positively contributing and medial metatarsals (first to third) negatively contributing. Previous research has demonstrated a medial shift in the center of pressure during the propulsive phase of gait (Hof et al., 2005). These findings suggest that a gait pattern characterized by enhanced lateral forefoot propulsion and foot eversion may facilitate medial pressure transfer, potentially improving propulsive efficiency and exhibiting greater plantar fascia stiffness. Cen et al., using finite element simulations, demonstrated that reducing the stiffness of the plantar fascia can effectively alleviate metatarsal conformity (Cen et al., 2023). Combined with previous research and the findings of this study, it can be concluded that metatarsal conformity is a key indicator of the biomechanical properties of the plantar fascia.

In summary, the characteristics of plantar pressure during walking are important indicators of the foot soft tissues condition. Adjustments to plantar pressure should consider the structural features of various parts of the foot to collaboratively regulate pressure distribution. This approach can optimize the mechanical properties of the foot soft tissue through appropriate insole design. Moreover, insights from studies on adaptive impedance control strategies in other fields can inform the optimization of resistance training devices or wearable assistive systems (Chen et al., 2024; Li et al., 2023). By modulating the state of plantar soft tissues in response to plantar kinetic characteristics, so as to enhance motor control capabilities and achieve superior rehabilitation outcomes.

## 5 Limitations

There are some limitations of the study. Firstly, the current modeling focused on plantar pressure and soft tissue conditions of young male individuals with normal foot during walking. Future studies are recommended to analyze different genders and age groups, focusing on abnormal foot types or pathological conditions such as flatfoot and diabetic foot, so as to establish an interpretable model incorporating movement performance for assessing risk of plantar soft tissue injury. Additionally, while the PSO-GA-BP regression network demonstrated favorable predictive performance in this study, it is still suggested that future research endeavors aim to increase the sample size, employing more precise algorithms and validation methods for models. This would not only further improve predictive accuracy but also enable more effective utilization of MIV algorithm to assess input layer impact on output layer, achieving interpretable modeling of plantar soft tissue mechanical condition based on different motions like running and jumping.

## 6 Conclusion

This study constructed an interpretable model of plantar soft tissue stiffness using plantar pressure across different regions during walking. The pressure features in the medial heel, midfoot area, and lateral mid-metatarsal regions during walking can better reflect plantar soft tissue stiffness. However, the mean pressure in the fourth metatarsal region demonstrated low test-retest reliability. Future studies should ensure measurement stability of this region and refine insole designs to mitigate plantar soft tissue fatigue in the specified areas.

## Data Availability

The raw data supporting the conclusions of this article will be made available by the authors, without undue reservation.
